# Selective Histopathology Examination for Circumcision Specimens: A Retrospective Observational Study

**DOI:** 10.7759/cureus.66151

**Published:** 2024-08-04

**Authors:** Baidar Khalabazyane, Rahel Rashid, Laura Mclaren, Roza Salah, Israa Kadhmawi, Joshua Philips

**Affiliations:** 1 Urology, Royal Bournemouth Hospital, Bournemouth, GBR; 2 General and Colorectal Surgery, Arrowe Park Hospital, Wirral, GBR; 3 Plastic and Reconstructive Surgery, Salisbury Foundation Trust, Bournemouth, GBR; 4 Medicine, Arrowe Park Hospital, Wirral, GBR

**Keywords:** foreskin histology, penile cancer, paraphimosis, phimosis, lichen sclerosus, balanitis xerotica obliterans (bxo), male circumcision

## Abstract

Background

Circumcision is a widely performed surgical procedure all over the globe. This can be for religious, cultural, or medical reasons. Routine histological examination of circumcision specimens is a standard practice in many healthcare systems, despite the relatively low incidence of premalignant or malignant lesions.

The primary objective of this study was to evaluate the necessity of routine histopathological examination of foreskin specimens following adult circumcision. Secondary objectives included determining the frequency of malignancy in these specimens, comparing malignancy rates between clinically suspicious and non-suspicious cases, and assessing the correlation between preoperative clinical suspicion and histopathological findings.

Aim

This study aimed to evaluate the necessity of routine histopathological evaluation for the foreskin after circumcision. We investigated the frequency of malignancy upon histopathological examination, in clinically suspicious cases compared to non-suspicious cases.

Method

A retrospective observational study was conducted at the Royal Bournemouth Hospital, analyzing data from 334 consecutive adult male patients who underwent circumcision between January 2012 and December 2016. The cohort was retrospectively divided into two groups: those with preoperative suspicious clinical features and those without it.

Clinical records on electronic patient records (EPR) were used for follow-up and to identify the percentage of malignancy after final histopathological examinations in both groups.

Results

Among the 334 patients, only nine patients (2.7%) were deemed as having suspicious clinical features preoperatively, of which, only three (0.9% of the total study sample) showed malignancy upon histological examination. The other six patients in this group were found to have balanitis xerotica obliterans (BXO). The other 325 patients (97.3%) were without clinically suspicious lesions preoperatively, and none were found to have any malignant lesions upon histopathological examination.

Conclusion

The low incidence of malignancy in circumcision specimens indicates that routine histological examination may not be essential for all cases. Among 334 samples, only three (0.9%) were malignant, and all were clinically suspected. Routine histopathological examination of the remaining 331 cases did not impact management or follow-up. Selectively submitting specimens for histology based on clinical suspicion could reduce opportunity costs and time, optimize resource allocation, and maintain appropriate diagnostic evaluation.

## Introduction

Circumcision is one of the most widely performed procedures worldwide. In the UK, a 2016 study estimated that around 20.7% of the male population were circumcised [1]. In 1948, the National Health Service (NHS) chose not to fund routine circumcision due to a lack of conclusive evidence of its medical efficacy [2]. Currently, the NHS funds circumcision only for medical reasons, covering about 1% of males. Additionally, around 5% of boys are circumcised for religious reasons [3].

In the majority of cases in the NHS, adult male circumcision has been restricted to purely medical indications. This could range from genital warts, benign and inflammatory conditions such as phimosis, and paraphimosis, to balanitis xerotica obliterans (BXO) and squamous cell carcinoma of the prepuce. Many surgeons habitually submit the excised prepuce for histological examination, citing concerns about the potential for unexpected malignancy.

Previous research has indicated that clinical diagnoses align with histological findings in more than 80% of cases and has rarely resulted in a change in management and that histological examination may be warranted only in cases where there is suspicion of malignancy [4,5].

Based on the literature and clinical experience, we posit that in cases with extensive disease, the prepuce will show distinctive morphological features that warrant clinicians to send for histopathological evaluation and future follow-up.

This study primarily aimed to assess the necessity of routine histopathological examination of foreskin specimens following adult male circumcision. Secondary aims included determining the frequency of malignancy in these specimens, comparing malignancy rates between clinically suspicious and non-suspicious cases, and evaluating the correlation between preoperative clinical suspicion and histopathological findings. We sought to explore the potential for a more selective histopathological examination approach based on preoperative clinical assessment, evaluate the cost-effectiveness of routine examinations, and identify other significant pathological findings.

## Materials and methods

A retrospective study was conducted at the Royal Bournemouth Hospital, analyzing data from 334 consecutive adult male patients who underwent circumcision between January 2012 and December 2016. Clinical records were obtained from the electronic patient records (EPR). Patients were followed up using the EPR system and looking for entries regarding similar complaints in the 7 years following the end of the study.

This study aimed to evaluate the histopathological findings in adult circumcision cases for malignancy and compare these findings with the predictive value of clinical examinations. We hypothesize, based on literature and clinical experience, that the prepuce in malignant cases exhibits distinctive morphological features, necessitating histopathological evaluation and follow-up. Conversely, histopathological analysis of a normal clinical examination is unlikely to alter management.

The cohort was divided into two groups: those with preoperative suspicious clinical features and those without it. The underlying criteria for “suspicious clinical features” were findings consistent with penile cancer: mainly painless ulcerative skin lesions, palpable nodule(s) on the penis, and inguinal lymphadenopathy.

Final histopathological evaluation was obtained for all (100%) of the sample cohort. As such, circumcision cases were excluded in cases below 16 years old, as well as non-medical indications of circumcision. No ethical concerns were raised. Initially a quality improvement project, the study received approval from the clinical governance board for data collection and presentation, followed by retrospective data analysis.

## Results

A cohort of 334 adult male circumcision cases was reviewed between January 2012 and December 2016. Hundred percent of the cases were included for histopathological analysis.

The cohort was divided into two groups: those with suspicious clinical features on preoperative clinical examination and those without any suspicion. The mean average age was 53 (± 21) years old, ranging from 16 years old to 98 years old. Only nine patients (2.7%) belonged to the group with preoperative suspicious findings, the rest (325 patients) belonged to the group with no suspicious clinical features for malignancy.

**Figure 1:**
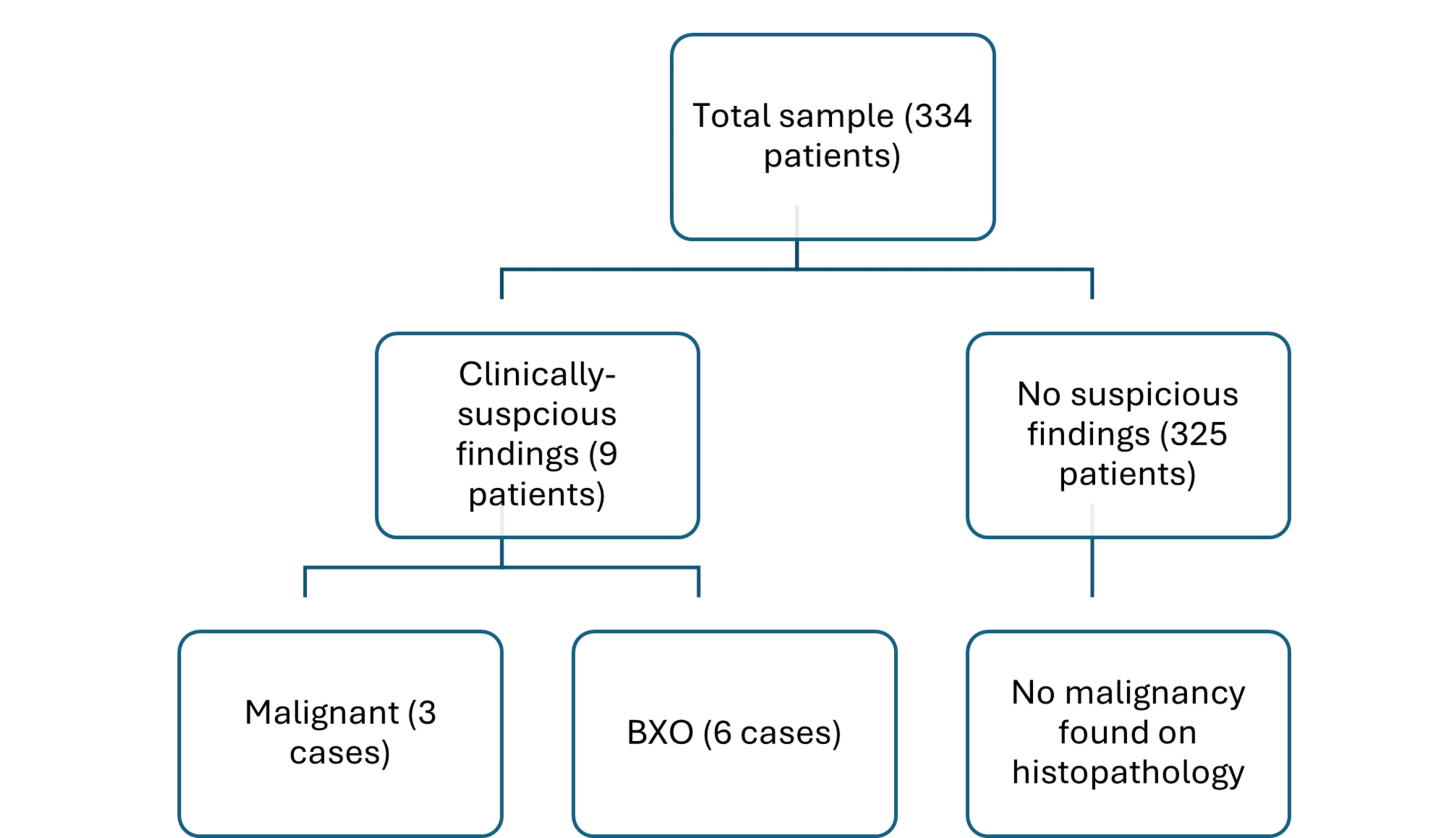
Final histological results BXO: balanitis xerotica obliterans

Of the first group (with suspicious clinical findings), three cases were positively diagnosed with malignancy (0.9% of the total sample, 33% of the group), and none of the cases had any inguinal lymphadenopathy. The rest of the group (n=6) showed BXO. From the second group, which were deemed without any suspicious clinical findings (n=325), none of the cases showed any malignant or dysplastic changes (Figure 1).

The histopathological evaluation resulted in a change in management in the three diagnosed cases of penile cancer; however, for the remaining 331 (99%) cases, it did not affect further management.

Indications for which the circumcision was performed (based on clinical examination) are detailed in Table 1.

**Table 1:** Overall clinical indications for which the circumcision was performed

Indication	Number of cases (%)
Phimosis	210 (62.8)
Balanitis xerotica obliterans	78 (23.3)
Ulceration	3 (0.9)
Thickening/nodule and erythema	6 (1.8)
Balanitis and recurrent infection	13 (3.9)
Paraphimosis	4 (1.2)
Zoon’s balanitis	9 (2.7)
Warts	3 (0.9)
Peyronie’s disease	2 (0.6)
Cysts	2 (0.6)
Other	4 (1.2)
Total	334

We have also correlated preoperative clinical findings with the final histopathological diagnosis which are summarized in Table 2.

**Table 2:** Percentage of correlation between clinical and histopathological diagnoses

Pathological diagnosis	% Correct clinical diagnosis
Balanitis xerotica obliterans	96
Squamous cell carcinoma	100
Zoon’s balanitis	50
Balanitis	40
Viral warts	100
Cysts	100

It’s noteworthy that while BXOs was reliably diagnosed clinically, other benign inflammatory conditions for example candidiasis, psoriasis, eczema, dermatitis lichen planus, and so on present greater diagnostic challenges. However, All three instances of penile cancer were accurately diagnosed through clinical examination.

## Discussion

Penile cancer is an uncommon malignancy, comprising less than 1% of male malignancies [6]. It is not confined to the foreskin, but can also arise on the prepuce, coronal sulcus, and/or glans as a painless mass, nodule, or ulcer. The literature consistently indicates that penile cancer, with its characteristic appearance, is typically identified clinically. Histological examination (punch biopsy, incisional, or excisional) should be requested for any lesion deemed suspicious [6,7].

We had 197 cases of histologically confirmed BXO and none of these has been re-referred in the previous 7 years of follow-up on our EPR system. The findings of this study highlight the potential for a more selective approach to histopathological examination of the foreskin after circumcision, which could lead to significant cost savings and more efficient resource allocation without compromising diagnostic accuracy. We have shown that clinical examination has an accurate positive predictive value and this is in concordance with the majority of the existing literature [8].

In the consensus of existing studies, preoperative diagnosis was found to be consistent with the final histology result, and histopathological examination had no significant impact on the management decision. Therefore, routine histological examination and outpatient follow-up are not recommended [8,9]. Moreover, studies have examined the re-referral rates of patients who undergo routine histology following circumcision compared to those who do not, and they found no difference [10].

Limitations of the study are expected. We have routinely sent for histopathology evaluation, and happily no unexpected findings were revealed, but we do concede that sometimes clinical examination for an early skin lesion may not appear suspicious at all and still be a potentially sinister pathology (e.g., intra-epithelial neoplasia). This calls for vigilant clinical examinations and follow-up. Furthermore, this is a single-center review only which restricts the available data sample. There is a need for further research and large collaborative studies to confirm these findings.

## Conclusions

The low incidence of malignancy in circumcision specimens suggests that routine histological examination may not be necessary for all cases. Out of 334 samples, only three (0.9%) cases were malignant, all of which were clinically suspected. Routine histopathological examination of the remaining 331 cases did not alter management or follow-up. Clinical diagnosis showed a high correlation with histopathological findings, especially for conditions like BXO and squamous cell carcinoma.

Selectively sending specimens for histology based on clinical suspicion could reduce opportunity costs and time, optimize resource allocation, and maintain appropriate diagnostic evaluation. However, the study acknowledges limitations, including the possibility of missing early, non-suspicious lesions. Further studies with larger sample sizes are needed to validate these findings.
